# Assessing a prediction model for depression risk using an early adolescent sample with self‐reported depression

**DOI:** 10.1002/jcv2.12276

**Published:** 2024-09-03

**Authors:** Eileen Y. Xu, Niamh MacSweeney, Gladi Thng, Miruna C. Barbu, Xueyi Shen, Alex S. F. Kwong, Liana Romaniuk, Andrew McIntosh, Stephen M. Lawrie, Heather C. Whalley

**Affiliations:** ^1^ Division of Psychiatry University of Edinburgh Royal Edinburgh Hospital Edinburgh UK

**Keywords:** adolescence, adolescent depression, major depressive disorder, prediction model, replication, risk factors, risk prediction

## Abstract

**Background:**

Major depressive disorder (MDD) in adolescence is a risk factor for poor physical and psychiatric outcomes in adulthood, with earlier age of onset associated with poorer outcomes. Identifying Depression Early in Adolescence Risk Score (IDEA‐RS) is a model for predicting MDD in youth aged >15 years, but replication in younger samples (<15 years) is lacking. Here, we tested IDEA‐RS in a younger sample (9–11 years) to assess whether IDEA‐RS could be applied to earlier onset depression.

**Methods:**

We applied IDEA‐RS predictor weights to 9854 adolescents (9–11 years) from the Adolescent Brain Cognitive Development (ABCD) Study, United States. We derived incident depression outcomes from self‐reported data at 2‐year follow‐up (11–13 years): incident MDD and increase in depression symptoms (DS). Sensitivity analyses were conducted using parent‐reported data. We assessed accuracy and calibration in predicting self‐reported incident depression and compared this to a refitted model with predictor weights derived in ABCD. Lastly, we tested associations between IDEA‐RS predictors and self‐reported incident depression.

**Results:**

External replication yielded better‐than‐chance discriminative capacity for self‐reported incident depression (MDD: AUC = 61.4%, 95% CI = 53.5%–69.4%; DS: AUC = 57.9%, 95% CI = 54.6%–61.3%) but showed poor calibration with overly extreme risk estimates. Re‐estimating predictor weights improved discriminative capacity (MDD: AUC = 75.9%, 95% CI = 70.3%–81.4%; DS: AUC = 64.8%, 95% CI = 61.9%–67.7%) and calibration. IDEA‐RS predictors ‘poorest level of relationship with the primary caregiver’ (OR = 4.25, 95% CI = 1.73–10.41) and ‘high/highest levels of family conflict’ (OR = 3.36 [95% CI = 1.34–8.43] and OR = 3.76 [95% CI = 1.50–9.38], respectively) showed greatest associations with self‐reported incident MDD.

**Conclusions:**

While IDEA‐RS yields better‐than‐chance predictions on external replication, accuracy is improved when differences between samples, such as case‐control mix, are adjusted for. IDEA‐RS may be more suited to research settings with sufficient data for refitting. Altogether, we find that IDEA‐RS can be generalisable to early adolescents after refitting and that family dysfunction may be especially impactful for this period of development.

**Key points**

- Identifying Depression Early in Adolescence Risk Score (IDEA‐RS) is a model that predicts incident depression in late adolescence (aged 15+ years).
- Here, we test IDEA‐RS in an early adolescent sample with self‐reported incident depression (aged 9–11 at baseline and 11–13 at follow‐up assessment).
- We find that, of the 11 predictors used in IDEA‐RS, poor relationships with the child’s primary caregiver and high family conflict have the greatest associations with incident depression.
- IDEA‐RS performs better than chance in predicting both youth‐ and parent‐reported incident depression, however refitting is required which may impact clinical utility.
- IDEA‐RS has now been replicated across six countries and shows promise as a method of stratifying depression risk in cohort studies for research purposes.

## INTRODUCTION

Major depressive disorder (MDD) is a widespread and leading cause of disability worldwide (GBD, 2019 Mental Disorders Collaborators, 2022). The prevalence of MDD rises from <1% to 4%–5% during adolescence and can lead to poorer psychological wellbeing, physical health and life satisfaction in adulthood (Clayborne et al., 2019; Schlack et al., 2021; Thapar et al., 2012; Thapar et al., 2022). Although the majority of adolescent depressive episodes remit within a year, risk of recurrence is high, with worse outcomes for those with chronic symptoms in adolescence (Johnson et al., 2018; Thapar et al., 2012). Earlier age of onset (<15 years) is also associated with longer illness duration, worse quality of life and increased odds of comorbid anxiety disorders, eating disorders and ADHD (Hammen et al., 2008; Weavers et al., 2021; Zisook et al., 2007). Together, this reinforces the clinical importance of identifying and supporting children and adolescents at increased risk of MDD, as a longer duration of untreated illness correlates with poorer treatment response (Ghio et al., 2014; Kraus et al., 2019).

Early identification of young people at increased risk of MDD could be instrumental in improving prognosis, providing opportunities for prevention and early intervention before MDD onset. To this end, numerous models aiming to predict depression onset have been developed based on established risk factors for MDD, frequently using machine learning (Botter‐Maio Rocha et al., 2021; Kwong et al., 2019; Toenders et al., 2022; Xiang et al., 2022). While performance of prediction models can appear excellent in training samples, external validations are imperative for assessing utility (Collins et al., 2024). Published prediction models for depression have performed well, with reported discriminative accuracy (ability to distinguish cases from controls, reported as AUC or C‐statistic) ranging from 72% to 90% for adolescent depression across 2–5 years (Botter‐Maio Rocha et al., 2021; Stephens et al., 2023; Toenders et al., 2022; Xiang et al., 2022), and as high as 96% in adults (Zulfiker et al., 2021), though external validations of models are limited.

One model which has been subject to external validation attempts is the IDEA‐RS (Identifying Depression Early in Adolescence Risk Score; IDEA‐RS) (Botter‐Maio Rocha et al., 2021). IDEA‐RS was originally trained in the 1993 Pelotas Birth Cohort study, using an a priori list of 11 predictors of incident MDD in late adolescence (18–19 years) using Penalised Maximum Likelihood Estimation. This sample consisted of *N* = 2192 adolescents aged 15 years at data collection with no prior history of depression. The 11 predictors identified fell into three categories: inherent characteristics (biological sex, skin colour), problematic behaviour (drug use, school failure, social isolation, fight involvement) and household dysfunction (poor relationship with mother, poor relationship with father, poor relationship between parents, childhood maltreatment, ran away from home).

IDEA‐RS performed well in the Pelotas study (C‐statistic = 0.78 [95% CI: 0.73–0.82]; equivalent to AUC) and was replicated in the UK E‐risk cohort (*N* = 1144; C‐statistic = 0.59 [95% CI: 0.55–0.63]; 12 years at baseline, 18 years at follow‐up) and the New Zealand Dunedin cohort (*N* = 739; C‐statistic = 0.63 [95% CI: 0.59–0.67]; 15 years at baseline, 18 years at follow‐up). Since then, IDEA‐RS has been applied to a cohort of Nepali former child soldiers and war‐affected civilians (*N* = 126; C‐statistic = 0.73 [95% CI: 0.62–0.83]; 11–18 years at baseline, 18+ years at follow‐up) (Brathwaite et al., 2021) a Nigerian adolescent sample (*N* = 1928; C‐statistic = 0.62 [95% CI: 0.58–0.66]; 14–16 years at baseline, follow‐up after 3 years) (Brathwaite et al., 2020), and the Great Smoky Mountains Study (*N* = 1209; C‐statistic = 0.63 [95% CI: 0.53–0.74]; 15 years at baseline, 19 years at follow‐up) (Caye et al., 2022). While these results suggest that this model may indeed be broadly generalisable across countries, previous replications have been limited by availability of predictors; only 7 of the 11 predictors were matched in the largest replication (Brathwaite et al., 2020). Both the Nigerian and E‐risk cohorts lacked data on family dynamics–the quality of relationships between child and caregivers, and relationships between caregivers–thus omitting a major facet of environmental risk for adolescents. A fuller replication of the IDEA‐RS in a large sample, with a full complement of matched predictors, may provide clearer insight into the generalisability of this model.

In the current study we applied IDEA‐RS to an early adolescent sample (*N* = 9856; aged 9–11 years at baseline and 11–13 years at MDD assessment). Similar to IDEA‐RS replications in Nigeria and Nepal (Brathwaite et al., 2020, 2021), we used questionnaire‐based, self‐reported MDD as the primary outcome in place of clinician‐diagnosed MDD; parent‐reported MDD was also used for comparison. Additionally, we included a secondary depression measure–increase in depressive symptoms (DS) over 2 years–to examine whether the IDEA‐RS is specific to predicting incident MDD. We were interested in (a) the reproducibility of the IDEA‐RS using all 11 predictors in predicting self‐reported incident MDD and (b) in the generalisability of the IDEA‐RS to a younger cohort for earlier detection of self‐reported MDD and increased DS 2 years later. Finally (c) since previous replications of IDEA‐RS varied substantially in model fit (AUC ranging from 0.59 to 0.73), we also examined the impact of model updating through recalibration (adjusting the baseline risk to match the new sample) and refitting (estimating new predictor weights in the new sample). Refitting derives new predictor weights from the sample data, thus allowing us to describe the strongest predictor variables for this younger sample which may be of future clinical interest. As refitted weights were derived through penalized logistic regression, where estimates are shrunk depending on the strength of association with the outcome, we also conducted exploratory analyses of these IDEA‐RS predictors using mixed‐effects logistic regression.

## MATERIALS AND METHODS

### Study description

The Adolescent Brain Cognitive Development (ABCD) study consists of *N* = ∼11,800 individuals aged 9–11 years at baseline assessment recruited from 21 sites in the United States (Garavan et al., 2018). Detailed descriptions of recruitment and data collection methods have been previously published (Casey et al., 2018; Feldstein Ewing et al., 2018; Garavan et al., 2018; Jernigan & Brown, 2018; Volkow et al., 2018). Participants gave informed consent (parent) or assent (child) and ABCD research sites are coordinated by a central Institutional Review Board, the ABCD Bioethics and Medical Advisory group and ABCD Steering Committee (Auchter et al., 2018; Clark et al., 2018). Data in this study were obtained from ABCD data release 4.0 (October 2021).

**FIGURE 1:**
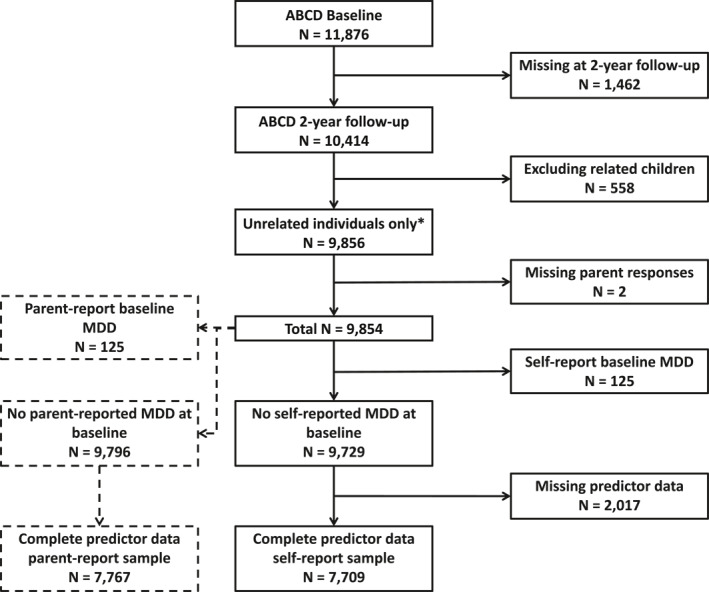
STARD diagram of included and excluded ABCD participants. ABCD, Adolescent Brain Cognitive Development study; MDD, Major Depressive Disorder; STARD, Standards for Reporting Diagnostic accuracy studies. *One participant ID was randomly sampled from each family ID to obtain a sample of unrelated participants. MDD case/control status was coded from youth self‐reported and parent‐reported responses to KSADS; no clinician diagnoses were obtained.

Participants were included in our analyses if they attended both the baseline and 2‐year follow‐up assessments (*N* = 10,414). We restricted the sample to unrelated individuals by selecting a random participant ID per family. Once youth self‐reported and parent‐reported MDD diagnoses were coded, MDD cases at baseline assessment were excluded. Finally, individuals with missing predictor data were excluded by listwise deletion (Figure 1).

In contrast to IDEA‐RS, we did not exclude participants based on low IQ (<70). Initial ABCD recruitment protocol screened participants for severe medical, sensory or neurological issues (including intellectual disability) that would impact the child’s ability to follow study procedures. Additionally, given the younger age of our sample, we did not exclude individuals without menarche.

### Measures

Predictor variables were selected from the battery of assessments at ABCD baseline (9–11 years) to match as closely as possible with the 11 predictors from IDEA‐RS. Due to data incompatibility between IDEA‐RS and ABCD, there were some discrepancies in the exact predictors selected despite best efforts to match variables as closely as possible (see Table 2 for details). Additionally, we selected youth self‐reported variables where available in ABCD for consistency with IDEA‐RS.

**TABLE 1:** Descriptive statistics for ABCD data by self‐reported incident MDD (Total *N* = 7709).

Demographics	Control (*N* = 7651)	Case (*N* = 58)
Age at baseline assessment	9.9 (0.61)	9.97 (0.63)
Age at 2‐year follow‐up	11.99 (0.65)	12.14 (0.7)
Household income:		
<50 K	1859 (26.3%)	21 (41.2%)
≥50 K & <100 K	2060 (29.2%)	17 (33.3%)
≥100 K	3145 (44.5%)	13 (25.5%)
Missing data	587 (7.7%)	7 (12.1%)
Parental education		
<HS diploma	315 (4.1%)	3 (5.2%)
HS diploma/GED	641 (8.4%)	4 (6.9%)
Some college	1872 (24.5%)	23 (39.7%)
Bachelor	2029 (26.6%)	12 (20.7%)
Postgraduate degree	2785 (36.4%)	16 (27.6%)
Missing data	9 (0.1%)	0 (0.0%)
IDEA‐RS predictors		
Female sex	3617 (47.3%)	40 (69.0%)
Ethnicity		
Black/African american	943 (12.3%)	11 (19.0%)
Hispanic/Latino	1157 (15.1%)	8 (13.8%)
White/Caucasian	4319 (56.5%)	24 (41.4%)
Other^a^	1232 (16.1%)	15 (25.9%)
Traumatic events		
One	1967 (25.7%)	18 (31.0%)
Multiple	675 (8.8%)	8 (13.8%)
School disengaged^b^	472 (6.2%)	4 (6.9%)
Complains of loneliness^c^	922 (12.1%)	14 (24.1%)
Gets in fights^d^	262 (3.4%)	4 (6.9%)
Runs away from home^e^	29 (0.4%)	1 (1.7%)
Ever tried alcohol/cigarettes/drugs^f^	1777 (23.2%)	14 (24.1%)
Relationship with caregiver 1		
Q1–Best	1600 (20.9%)	6 (10.3%)
Q2–Very good	1547 (20.2%)	9 (15.5%)
Q3–Good	1568 (20.5%)	13 (22.4%)
Q4–Poor	1498 (19.6%)	12 (20.7%)
Q5–Poorest	1438 (18.8%)	18 (31.0%)
Relationship with caregiver 2		
Q1–Best	1540 (20.1%)	8 (13.8%)
Q2–Very good	1536 (20.1%)	10 (17.2%)
Q3–Good	1545 (20.2%)	14 (24.1%)
Q4–Poor	1521 (19.9%)	12 (20.7%)
Q5–Poorest	1509 (19.7%)	14 (24.1%)
Family conflict		
Q1–Least	1633 (21.3%)	5 (8.6%)
Q2–Little	1609 (21.0%)	10 (17.2%)
Q3–Moderate	1531 (20.0%)	13 (22.4%)
Q4–Much	1511 (19.7%)	14 (24.1%)
Q5–Most	1367 (17.9%)	16 (27.6%)

Abbreviations: DS, Depression Symptoms; GED, General Educational Development; HS, High School; MDD, Major Depressive Disorder.

^a^ Any ethnicities with fewer than five observations in a single category have been included as “Other” to preserve anonymity of participants. “Other” includes: Afro‐Caribbean/Indo‐Caribbean/West Indian, American Indian/Alaska Native, East Asian, Eastern European, Middle Eastern/North African, Mixed ethnicity, Native Hawaiian or Pacific Islander, South Asian, Southeast Asian, Western European, None, Other ethnicity.

^b^ “School bores me” AND “good grades aren’t important to me” endorsed.

^c^ Child complains of loneliness/would rather be alone (sometimes or often), not true = 0.

^d^ Child gets in many fights (sometimes or often), not true = 0.

^e^ Child runs away from home (sometimes or often), not true = 0.

^f^ Combining responses about any use of alcohol, tobacco (in any format), cannabis and other illicit substances. Dichotomised to never tried (0) or ever tried (1).

**TABLE 2:** Predictors of MDD in ABCD, as matched from the IDEA‐RS training sample.

Variable (reference group)	IDEA‐RS description (Pelotas birth cohort; youth self‐report)	ABCD description
Sex (M)	Self‐reported sex	Sex at birth (M/F)
Ethnic group^a^ (white)	Self‐assigned skin colour (white/non‐white)	MEIM: White (0)/non‐white (1)^b^
Traumatic events^d^ (none)	Lifetime psychological, physical and sexual abuse and/or neglect at age 15; zero positive answers = none, 1 positive = probable, 2 or more = severe	KSADS traumatic events: 0 = none; 1 = one event; 2 = multiple events
School disengagement (0)	“Have you ever been retained in school (to repeat the same school year)?” was classified as “failing at school” = 1; otherwise = 0	1 = SRPF: “School bores me” AND “good grades aren’t important to me” endorsed, otherwise = 0
Loneliness^d^ (0)	“Do you normally meet up with friends to chat, play or do other things? If YES, how many days in a given week?”	1 = CBCL: Child complains of loneliness/would rather be alone (sometimes or often), not true = 0
Fights involvement^d^ (0)	“In the last year, have you ever gotten into a physical fight that someone got hurt?”	1 = CBCL: Child gets in many fights (sometimes or often), not true = 0
Ran away from home^d^ (0)	“Have you ever run away from home?”	1 = CBCL: Child runs away from home (sometimes or often), not true = 0
Ever tried alcohol, cigarettes or drugs (0)	Dichotomous variable combining responses to dichotomous questions about any lifetime use of alcohol, tobacco, cannabis, cocaine and inhalants; any positive answer = “1”; otherwise = “0”	YSU: Combining responses about any use of alcohol, tobacco (in any format), cannabis and other illicit substances. Dichotomised to never tried (0) or ever tried (1)
Relationship with caregiver 1 [who accompanied child to appointment] (1, best)	“How do you rate your relationship with your mother?”; choices of answers: great = 1, very good = 2, good = 3, regular = 4, bad = 5; inserted as a categorical variable into the model	CRPBI: Continuous summary score of caregiver acceptance, separated into quintiles named as 1 = best, 2 = very good, 3 = good, 4 = poor, 5 = poorest.
Relationship with caregiver 2 (1, best)	“How do you rate your relationship with your father?”; choices of answers: great = 1, very good = 2, good = 3, regular = 4, bad = 5; inserted as a categorical variable into the model	As above
Family conflict (1, least conflict)	“How do you rate the relationship between your father and your mother?”; choices of answers: great = 1, very good = 2, good = 3, regular = 4, bad = 5; inserted as a categorical variable into the model	FES: Continuous summary score of family dynamics, cohesion, expressiveness and conflict, separated into quintiles named as 1 = least, 2 = little, 3 = moderate, 4 = much, 5 = most.
Incident MDD^c^	Depression diagnosis at age 18: Evaluation of major depressive episode diagnosis with DSM‐IV‐TR criteria in the previous 2 weeks	MDD present at 2‐year follow‐up (11–13 years), with no MDD at baseline assessment (9–11 years)
Increase in severity (additional MDD outcome)^c^		Increase in depression symptoms between baseline (9–11 years) and 2‐year follow‐up (11–13 years).

*Note*: ABCD variables are all youth‐reported unless indicated otherwise.

Abbreviations: ABCD, Adolescent Brain Cognitive Development study; CBCL, Child Behaviour Checklist, Achenbach System of Empirically Based Assessment (ASEBA); CRPBI, Children’s Report of Parental Behavioural Inventory; DSM‐IV‐TR, Diagnostic and Statistical Manual of Mental Disorders, fourth edition, text revision; DSM‐V, Diagnostic and Statistical Manual of Mental Disorders, fifth edition; FES, Family Environment Scale‐Family Conflict Subscale Modified from PhenX; IDEA‐RS, Identifying Depression in Early Adolescence Risk Score; KSADS, Kiddie Schedule for Affective Disorders and Schizophrenia; MDD, Major Depressive Disorder; MEIM, Parent Multi‐Group Ethnic Identity‐Revised Survey; SRPF, School Risk and Protective Factors Survey; YSU, Youth substance use interview.

^a^ Although a youth‐reported version of ethnicity was available, parent‐reported ethnicity was assessed due to the quantity of missing data (N missing: Youth = 4770; Parent = 197).

^b^ We used a binary measure of race here in line with the original IDEA‐RS study so that our results can be comparable, however the authors acknowledge that binary categories are reductive and cannot accurately describe the diverse lived experiences of cultural and ethnic experiences associated with skin colour. The “non‐white” group contains the following ABCD descriptions: None, American Indian/Alaska Native, Middle Eastern/North African, Native Hawaiian or Pacific Islander, Mixed ethnicity, Other ethnicity, Western European, Eastern European, Hispanic/Latino, Black/African American, Afro‐Caribbean/Indo‐Caribbean/West Indian, East Asian, South Asian, Southeast Asian.

^c^ Indicates a variable with both youth and parent‐reported responses.

^d^ Indicates a variable with only parent‐reported response available.

We used self‐reported measures of incident depression, as clinical diagnostic interviews were not conducted in ABCD. Self‐reported lifetime MDD cases were coded from the computerised Kiddie Schedule for Affective Disorders and Schizophrenia, version 5 (KSADS‐COMP) by combining current (past 2 weeks) and past depression symptoms (Barch et al., 2021; Kaufman et al., 1997). This was delivered at baseline (9–11 years) and at 2‐year follow‐up (11–13 years). Due to data error in ABCD release 4.0, diagnoses were coded by mapping KSADS questions onto symptom criteria in the Diagnostic and Statistical Manual of Mental Disorders fifth Edition (DSM‐5), detailed in the Supplementary Materials. Irritable mood was included as an alternative to the core symptom of depressed mood, as suggested in the DSM‐5 for children and adolescents (American Psychiatric Association, 2013). Incident cases were identified as participants with no depression history at baseline who self‐reported MDD at 2‐year follow‐up (Figure 1).

An additional measure of depression symptoms (DS) was encoded based on the same self‐reported KSADS data (Shen et al., 2021). “Mild” DS was characterised as five to six total symptoms, or at least one core and one secondary symptom. “Moderate” DS was characterised as seven to eight total symptoms or at least one core and two secondary symptoms. “Severe” DS was characterised as both core symptoms and 3+ secondary symptoms, or as a clinical case with suicidal thoughts or behaviour. Core and secondary symptoms for MDD/DS are listed in the supplementary materials. Self‐reported increased DS cases were defined as participants with at least a one‐step increase (e.g. control to mild) in DS at 2‐year follow‐up, compared to baseline.

### Statistical analyses

IDEA‐RS coefficients were applied to ABCD data to derive predictive values for each participant on the logit scale; these were then back‐transformed to obtain estimates on the probability scale. To assess prediction of incident self‐reported MDD, we compared metrics of discrimination (Area under the curve (AUC), often known as the C‐statistic for binary outcomes), overall fit (Brier score), and calibration (calibration‐in‐the‐large, calibration slope). Additionally, we fit calibration curves to compare IDEA‐RS predicted probabilities with observed probabilities of incident self‐reported depression in ABCD.

Next, we updated the IDEA‐RS model using ABCD data by re‐estimating elements of the linear predictor in a two‐stage approach. This improves calibration and accounts for differences in case‐control mix between development and validation studies. However, model updating can also result in systematic issues of overfitting (Binuya et al., 2022).

First, we re‐estimated the intercept, leaving predictor coefficients unchanged (“recalibration”). This updates the baseline risk to reflect the proportion of cases and controls in ABCD, improving calibration issues that stem from differences between development and validation samples. To achieve this, we fit a logistic regression model with the IDEA‐RS linear predictor (minus the intercept) as an offset variable (fixed slope of 1). After assessing the recalibrated model, we fully refit the IDEA‐RS model by re‐estimating all predictor weights. Following the original method used for IDEA‐RS, we entered all predictors and their interactions into a logistic regression with penalised maximum likelihood estimation (PMLE) (Botter‐Maio Rocha et al., 2021). Penalty terms used in refitting were derived separately for youth self‐reported incident MDD and parent‐reported incident MDD, with the final penalty term defined as the median value across 1000 bootstrapped model comparisons. Additional detail on penalty selection is available in the Supplementary Materials.

Since predictor weights obtained through penalized models are shrunk closer to zero depending on the strength of their association with the outcome, we tested associations between the 11 predictor variables and incident self‐reported MDD with a mixed‐effects logistic regression. Random intercepts were included to account for nesting in ABCD data.

For self‐reported increased DS, analyses were similar. As we were interested in the generalisability of IDEA‐RS to this new outcome measure, we did not make any changes to the above methodology to accommodate this new outcome. Thus, IDEA‐RS predicted probabilities of incident MDD were compared against observed probabilities of self‐reported increased DS, with performance measured by the same metrics. Model updating was not performed for self‐reported increased DS since the aim was to assess generalizability of IDEA‐RS predictors and not to produce a model predicting DS. We did, however, compare updated (self‐reported) incident MDD predictions against observed probabilities of self‐reported increased DS. Additionally, we compared associations between IDEA‐RS predictors and self‐reported increased DS through mixed‐effects logistic regression models.

Although in‐depth descriptions of model performance are available elsewhere (Riley et al., 2024; Steyerberg & Vergouwe, 2014) we provide a brief summary of each metric used to aid interpretation:

Area under the receiver‐operating‐characteristic (ROC) curve (AUC) is analogous to the C‐statistic for binary outcomes and measures discriminative accuracy (distinguishing cases from controls). An AUC of 50% indicates performance no better than chance; an AUC of 100% indicates perfect discrimination.

Brier score (Brier, 1950) is a measure of overall fit akin to mean squared error. This compares observed outcomes to predicted probabilities, with a value of 0 indicating a perfect fit.

Calibration‐in‐the‐large and calibration slope are quantified by plotting observed outcomes against predicted probabilities. Calibration‐in‐the‐large (intercept) values closer to 0 indicate better overall agreement between predicted probabilities and actual risk. Calibration slope describes extremeness ‐ values <1 indicate overly extreme predictions (i.e. predictions closer to 1 are too high, predictions closer to 0 are too low); values >1 indicate the opposite (Steyerberg & Vergouwe, 2014; Van Calster et al., 2019).

### Sensitivity analyses

Sensitivity analyses were performed using parent‐reported depression and are reported in the Supplementary Materials. While prevalence of youth‐reported incident MDD was low (0.8%) in ABCD, prevalence was lower for parent‐reported incident MDD (0.6%). Therefore, we prioritise self‐reported results in this manuscript for consistency with the original IDEA‐RS.

## RESULTS

Descriptive statistics of the ABCD sample by self‐reported incident MDD are presented in Table 1. A summary of the IDEA‐RS predictor variables and MDD measures selected in ABCD is presented in Table 2. *N* = 68 participants were identified as self‐reported incident MDD cases, with *N* = 3 overlapping with parent‐reported incident cases (Cohen’s *κ* = 0.048; total parent‐reported = 52; Table S1). *N* = 365 participants were categorized as self‐reported increased DS cases; *N* = 8 of these overlapped with parent‐reported incident MDD cases, *N* = 42 overlapped with parent‐reported increased DS cases (Cohen’s *κ* = 0.078; total parent‐reported = 373; Table S2).

### Prediction of self‐reported incident MDD

**FIGURE 2:**
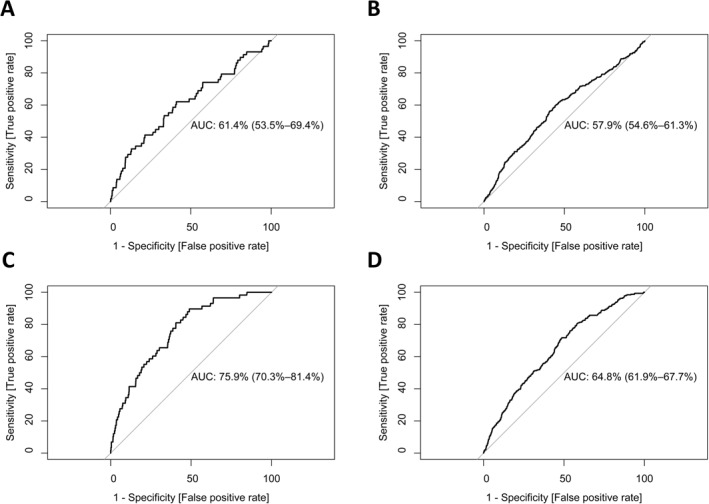
Receiver operating characteristic (ROC) curves for (A) external replication of IDEA‐RS in ABCD with incident MDD as an outcome; (B) external replication with increased DS as an outcome; (C) ABCD refitted model of incident MDD as an outcome and (D) ABCD refitted model with increased DS as an outcome. The 45‐degree diagonal indicates a classifier with no predictive value; classifiers with near‐perfect discrimination are indicated by curves close to the top‐left corner. ABCD, Adolescent Brain Cognitive Development study; AUC, Area Under the Curve; IDEA‐RS, Identifying Depression in Early Adolescence Risk Score.

**FIGURE 3:**
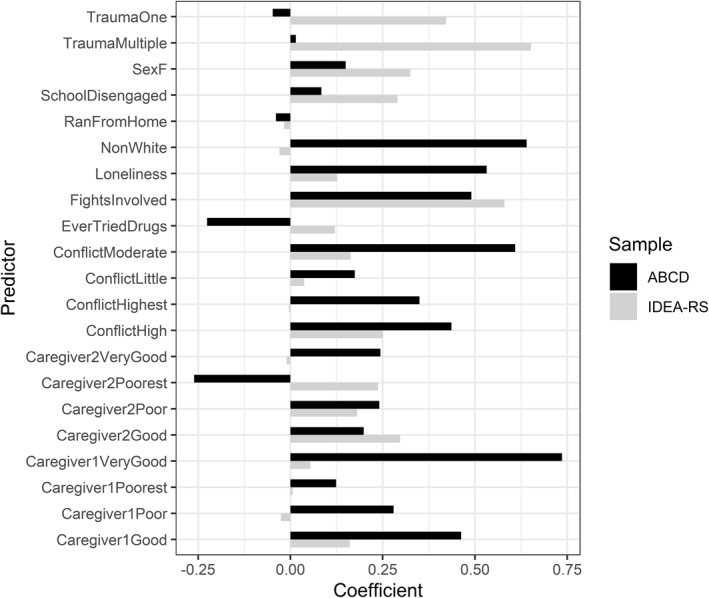
Coefficients from the refitted prediction model (ABCD refitted) compared to coefficients from IDEA‐RS. ABCD, Adolescent Brain Cognitive Development study; Caregiver 1, primary caregiver; Caregiver 2, secondary caregiver; IDEA‐RS, Identifying Depression in Early Adolescence Risk Score.

IDEA‐RS showed lower discriminative capacity for self‐reported incident MDD in ABCD (ABCD: AUC = 61.4%, 95% CI = 53.5%–69.4%; IDEA‐RS: AUC = 77.8%, 95% CI = 75.6%–79.9%) (Figure 2A). External validation also showed poor calibration and overly extreme risk estimates (Table 3, Supplementary Figure S1), though overall fit in ABCD (Brier score = 0.008) was better than the published Brier score for IDEA‐RS (0.03). After recalibrating IDEA‐RS by re‐estimating the model intercept, there was a slight improvement in overall fit (Δ Brier score = −0.001) and calibration in‐the‐large (Δ Calibration‐in‐the‐large = 0.79), but no change in discriminative capacity or calibration slope (Table 3). Fully refitting IDEA‐RS improved discriminative capacity (Δ AUC = 14.5%; Refitted AUC = 75.9%, 95% CI = 70.3%–81.4%) and further improved calibration (Refitted calibration‐in‐the‐large = 0.87), though overall risk estimates became overly conservative (refitted calibration slope = 1.19). No improvement was observed for model fit (Table 3). Of the 11 IDEA‐RS predictors, only non‐white ethnicity and poorest relationship with the primary caregiver had significant coefficients in the refitted model (Figure 3).

**TABLE 3:** Fit statistics for replication and refitting of IDEA‐RS in ABCD.

	IDEA‐RS		ABCD (external validation)		ABCD (intercept recalibration)		ABCD (fully refitted)	
	Incident MDD	Increase DS	Incident MDD	Increase DS	Incident MDD	Increase DS	Incident MDD	Increase DS
AUC (95% CI)	77.8% (75.6%–79.9%)	‐	61.4% (53.5%–69.4%)	57.9% (54.6%–61.3%)	61.4% (53.5%–69.4%)	57.9% (54.6%–61.3%)	75.9% (70.3%–81.4%)	64.8% (61.9%–67.7%)
Brier score	0.03	‐	0.008	0.04	0.007	0.04	0.007	0.04
Calibration in‐the‐large	0.00	‐	−2.28	−1.49	−1.49	−1	0.87	0.21
Calibration slope	1.26	‐	0.69	0.43	0.69	0.43	1.19	0.67

*Note*: AUC: area under the curve, equivalent to C‐statistic for binary outcomes. Values closer to 100% indicate better discriminative accuracy. Brier score: quadratic scoring rule that combines calibration and discrimination; a score of 0 indicates perfect overall fit. Calibration in‐the‐large: model intercept; results closer to 0 indicate better overall agreement between predicted probabilities and observed probabilities. Calibration slope: values <1 indicate overly extreme model predictions, values >1 indicate the opposite. A calibration slope of one indicates perfect calibration.

Abbreviations: ABCD, Adolescent Brain Cognitive Development study; DS, Depression Symptoms; IDEA‐RS, Identifying Depression in Early Adolescence Risk Score; MDD, Major Depressive Disorder.

Seven predictors were found to significantly associate with self‐reported incident MDD at 2‐year follow‐up. Greatest odds ratios were seen for poorest level of relationship with the primary caregiver (Odds ratio; OR: 4.25; 95% CI: 1.73–10.41; compared to excellent). Two household dysfunction measures had comparatively large odds ratios: highest and high levels of family conflict (OR_Highest_: 3.76, 95% CI: 1.50–9.38; OR_High_: 3.36, 95% CI: 1.34–8.43; compared to none). Female sex (OR: 2.34, 95% CI: 1.41–3.90), non‐white ethnicity (OR: 2.00, 95% CI: 1.22–3.28) and parent‐reported child loneliness (OR: 2.51, 95% CI: 1.46–4.32) were also associated with increased odds of self‐reported incident MDD at 2 years (Table 4).

**TABLE 4:** Mixed‐effects logistic regression of incident MDD and increase in depressive symptoms using 11 ABCD variables matched to the IDEA‐RS training sample.

Predictor	Reference	Incident MDD: OR (95% CI)	Increase in DS: OR (95% CI)
Female sex	*Male*	2.34 (1.41–3.90)**	1.94 (1.56–2.40)***
Non‐white ethnicity	*White*	2.00 (1.22–3.28)**	1.67 (1.33–2.09)***
Trauma (one)	*None*	1.58 (0.93–2.70)	1.18 (0.93–1.51)
Trauma (multiple)		2.21 (1.12–4.37)*	1.58 (1.15–2.18)**
School disengagement	*No*	1.74 (0.79–3.83)	1.02 (0.67–1.56)
Loneliness	*No*	2.51 (1.46–4.32)***	2.02 (1.56–2.61)***
Fights involvement	*No*	2.10 (0.84–5.26)	1.74 (1.13–2.69)*
Ran away from home	*No*	3.70 (0.50–27.56)	1.93 (0.59–6.34)
Ever tried alcohol/tobacco/drugs	*No*	1.18 (0.69–2.04)	0.89 (0.69–1.15)
Caregiver 1: Poorest	*Excellent*	4.25 (1.73–10.41)**	1.94 (1.37–2.74)***
Caregiver 1: Poor		2.45 (0.94–6.38)	1.63 (1.14–2.33)**
Caregiver 1: Good		2.56 (0.99–6.62)	1.74 (1.22–2.48)**
Caregiver 1: Very good		1.55 (0.55–4.35)	1.11 (0.76–1.64)
Caregiver 2: Poorest	*Excellent*	1.78 (0.75–4.25)	1.86 (1.32–2.63)***
Caregiver 2: Poor		1.51 (0.62–3.69)	1.24 (0.85–1.80)
Caregiver 2: Good		1.74 (0.73–4.14)	1.33 (0.92–1.92)
Caregiver 2: Very good		1.25 (0.49–3.16)	1.00 (0.68–1.48)
Family conflict: Little conflict	*None*	1.68 (0.61–4.62)	1.09 (0.75–1.56)
Family conflict: Moderate		2.26 (0.86–5.96)	1.34 (0.95–1.91)
Family conflict: High conflict		3.36 (1.34–8.43)**	1.67 (1.19–2.34)**
Family conflict: Highest conflict		3.76 (1.50–9.38)**	1.42 (1.00–2.01)

*Note*: * uncorrected *p* < 0.05; ** uncorrected *p* < 0.01; *** uncorrected *p* < 0.001. Results in bold font indicate statistical significance after FDR correction for multiple testing.

Abbreviations: Caregiver 1, primary caregiver; Caregiver 2, secondary caregiver; MDD, Major Depressive Disorder; OR, odds ratio.

### Prediction of self‐reported increased DS

IDEA‐RS yielded better‐than‐chance discriminative capacity for self‐reported increased DS (AUC = 57.9%, 95% CI = 54.6%–61.3%), but showed poor calibration and overly extreme risk estimates (Table 3). Despite the change in outcome, model fit for prediction of increased DS (Brier score = 0.04) was comparable to the original IDEA‐RS (Brier score = 0.03). Adjusting the IDEA‐RS intercept to fit ABCD incident MDD data also improved calibration‐in‐the‐large for increased DS (Δ Calibration‐in‐the‐large = 0.49). Fully refitting IDEA‐RS to self‐reported incident MDD also improved discriminative capacity for increased DS (Δ AUC = 6.9%; Refitted AUC = 64.8%, 95% CI = 61.9%–67.7%) and calibration‐in‐the‐large (Refitted calibration‐in‐the‐large = 0.21, refitted calibration slope = 0.67).

Female sex, non‐white ethnicity, parent‐reported child loneliness, experience of multiple traumatic events and involvement in fights were associated with increased DS at 2 years (OR range: 1.58–2.02). In terms of household dysfunction, poorest, poor and good (but not very good) relationships with the child’s primary caregiver were associated with self‐reported increased DS, as was poorest relationship with the child’s secondary caregiver and high levels of family conflict (OR range: 1.63–1.94) (Table 4).

### Sensitivity analyses

Results for parent‐reported MDD were similar. IDEA‐RS showed better‐than‐chance discriminatory accuracy for parent‐reported incident MDD (AUC = 63.2%; 95% CI = 54.8%–71.6%) which improved further upon full refitting (AUC = 86.9%; 95% CI = 81.9%–91.9%). For parent‐reported increased DS, refitting only improved discriminatory accuracy slightly (external validation: AUC = 56.5%, 95% CI = 53.2%–59.8%; refitted: AUC = 61.1%, 95% CI = 57.8%–64.4%). Full results are present in the supplementary materials (Tables S3–S4, Figures S2‐S4).

## DISCUSSION

In this replication study, we aimed to fit a prediction model of adolescent MDD developed in Brazil to an American sample of early adolescents with self‐reported MDD outcomes after 2 years. IDEA‐RS performed better‐than‐chance at discriminating incident cases from controls when the original predictor weights were used, however the model produced overly extreme estimates of risk (lower risk predicted as too low, higher risk predicted as too high). Both discriminative capacity and calibration of risk estimates improved substantially after predictor weights were re‐estimated in ABCD, with discriminative capacity similar to that of the original IDEA‐RS results (Botter‐Maio Rocha et al., 2021). This indicates that IDEA‐RS is capable of producing more accurate estimates when differences between samples, such as case‐control mix, are adjusted for. These results are consistent with findings from previous external replications, though the degree of improvement on refitting varies; this study shows the highest discriminative capacity across existing IDEA‐RS validation studies (Botter‐Maio Rocha et al., 2021; Brathwaite et al., 2020; Brathwaite et al., 2021). Our results indicate that the IDEA‐RS can be generalised, with refitting, to a younger sample than the model was initially trained in.

In terms of predicting future worsening, we tested the model’s ability to predict an increase in self‐reported DS over 2 years. Prediction of self‐reported increased DS was better‐than‐chance when IDEA‐RS predictor weights were used; this improved further when predictor weights from ABCD data were used. This refitted model performed well in terms of overall agreement between estimated and observed risk, outperforming incident MDD in this regard, and showed less extreme risk estimates compared to the original IDEA‐RS predictions.

The a priori risk factors selected by Botter‐Maio Rocha et al. (2021) may also differ in pertinence to MDD risk in younger American adolescents. We found that predictors ‘school disengagement’, ‘involvement in fights’, ‘ran away from home’ and ‘ever trying drugs or alcohol’ did not associate with self‐reported incident MDD or increased DS in our sample. These differences could be a result of age differences between the IDEA‐RS training sample (aged 15 years) and ABCD (aged 9–11 years), and/or broad socioeconomic differences (e.g. education, income) between Brazil, an upper‐middle income country and the US, a high income country.

Our results show better discriminatory capacity for parent‐reported MDD compared to youth self‐reported MDD. This mirrors the stronger associations found between parent‐reported depression measures and brain structure found by Shen et al. (2021) in ABCD. Integrating reports from multiple informants in the diagnosis of adolescent psychopathology can prove challenging, particularly in internalising problems such as depression where reporter discrepancy is greatest (De Los Reyes et al., 2015; Martel et al., 2017). A comparison of KSADS‐COMP parent and youth‐reported MDD in 6‐18 year‐olds showed greater agreement in identifying individuals with no MDD (89% agreement), but lower agreement for individuals with MDD (43%), though actual frequencies of MDD diagnoses were comparable (Townsend et al., 2020). We observed a similar pattern in ABCD with only a small overlap between parent‐ and youth‐reported incident MDD cases (*N* = 3; Table S1); this may also reflect differences in endorsing depression symptoms as correspondence between youth‐ and parent‐reported increased DS was also low (*N* = 48; Table S2).

We observed a lower prevalence of self‐reported depression in ABCD (0.8%) compared to other replication studies, where prevalence ranged from 3.1% (Caye et al., 2022) to 17.7% (Botter‐Maio Rocha et al., 2021). This may be due to the younger age of the ABCD cohort, but also resembles the 1.3% prevalence rate observed in children and adolescents in high‐income countries (Barican et al., 2022). We note that depression at age 11–13 is relatively rare and hard to predict, with early signs of depression potentially being manifestations of other types of psychopathology that were not accounted for. This lack of specificity may have limited the performance of IDEA‐RS, especially as it yielded overly extreme risk estimates. Similar results were seen with increased DS, a broader phenotype of worsening symptoms, which might suggest that in early adolescence, IDEA‐RS predictions relate more to an overall risk of future psychopathology.

Despite the lower prevalence, we considered it an important aspect of the analysis to look at this early period given the value of early prediction and intervention in depression risk in reducing the long‐term impacts of depression in adolescence, particularly with the move to a broader definition of adolescence being between 10 and 24 years of age (Thapar et al., 2022). As the ABCD sample ages and more longitudinal data becomes available, the testing of IDEA‐RS in this study could form the groundwork for future assessments of IDEA‐RS performance regarding time to MDD onset, providing a potential window where MDD risk could be assessed and mitigated.

The IDEA‐RS is, to date, the prediction model with the most external validation attempts and shows consistency in distinguishing cases from controls in new samples. IDEA‐RS performance in new samples–AUC of 72.9% after re‐estimating model parameters in ABCD–is comparable to other less‐validated models of adolescent depression such as those by Stephens et al. (2023) (72.2% on external validation), Toenders et al. (2022) (68%–72% on external validation) and Xiang et al. (2022) (77%–90%, no external validation). IDEA‐RS shows lower discriminative capacity compared to prediction models for other adolescent mental health‐related outcomes such as overall mental health problems (74%) (Tate et al., 2020), suicidal thoughts (86%) (Czyz et al., 2023) and non‐suicidal self‐injury (83%) (Zhong et al., 2024).

Although methodology and selection of predictor variables differed between models, both Stephens et al. (2023) and Toenders et al. (2022) include sex and stressful life events as predictors of future MDD. In particular, Toenders et al. (2022) took a feature reduction approach and identified a composite measure of stressful life events which included family conflict as a significant predictor. Given this, and the similarity of performance in external validation despite being developed in different regions–Brazil (Botter‐Maio Rocha et al., 2021), 8 countries across Europe (Toenders et al., 2022) and the United Kingdom (Stephens et al., 2023)—this might indicate that these models’ discriminative capacity rely to some degree on a strong influence from biological sex and stressful life events (particularly in the family).

When compared to clinical risk prediction models for cardiovascular disease with discrimination C‐statistics of 0.86 (similar to AUC = 86%) and above (Hippisley‐Cox et al., 2017), it is clear that clinical utility of IDEA‐RS would be limited. Despite this, IDEA‐RS might remain useful in research contexts, such as for quantifying depression risk in cohort studies where the algorithm can be refitted–it has since been used for this purpose in the E‐Risk study (Latham et al., 2022). As longitudinal cohorts such as ABCD pass through peak ages of MDD onset, IDEA‐RS could be a valuable starting point for establishing a timeframe where later MDD risk could be more accurately predicted. With further longitudinal data, IDEA‐RS may also give insight into the relevance of specific predictors in measuring MDD risk over the lifespan.

### Strengths and limitations

The primary limitation of this study is the lack of clinically‐ascertained diagnoses of MDD in ABCD. While expert diagnosis through semi‐structured or fully structured interviews following a standard manual such as the DSM or ICD is the gold‐standard for identifying MDD, this is often impractical for large studies such as ABCD (Kiely & Butterworth, 2015). This reduces comparability of our results to those from the original IDEA‐RS publication (cohorts in Brazil, UK, New Zealand) and makes it difficult to assess clinical use, though other published IDEA‐RS replications have also used self‐reported and computer algorithm‐assessed measures of MDD (Nepal, Nigeria, US) (Botter‐Maio Rocha et al., 2021; Brathwaite et al., 2020; Brathwaite et al., 2021; Caye et al., 2022). Using self‐reported measures of MDD did, however, allow us to test IDEA‐RS in not only a younger sample, but also a larger population‐based sample than would otherwise be possible.

We also note that agreement between self‐report and parent‐report MDD measures was very low in ABCD, which should be considered when interpreting our results–poor discrimination between MDD cases and controls could indicate low confidence in the MDD outcome itself in addition to differences in MDD risk. As differences in parent‐report and self‐reported depression have been reported previously in ABCD (Shen et al., 2021), further data releases could offer opportunities for longitudinal investigation of reporter discrepancies in this cohort. On this basis, future investigation of MDD risk in the ABCD cohort could involve comparisons between models trained on self‐reported and parent‐reported data and their predictive capacity for clinical diagnoses of MDD in late adolescence or early adulthood, closer to the peak age of MDD onset.

While we attempted to match all 11 IDEA‐RS predictors to the ABCD study, this was limited by variable availability in ABCD. Namely, the predictors ‘relationship with mother’, ‘relationship with father’ and ‘relationship between parents’ were measured on a five‐point Likert scale in the IDEA‐RS training sample; for this study we split scores from the Parental Behaviour Inventory’s Care subscale and Family Environment Scale‐Family Conflict Subscale into quintiles to match the original predictor structure. While this created a 5‐level variable for which IDEA‐RS weights could be used, the use of quintiles results in a lack of a meaningful reference point for these variables. Similarly, the IDEA‐RS measure of ‘school failure’ could not be accurately matched to ABCD–the ‘school disengagement’ variable used can only serve as a proxy. Additionally, IDEA‐RS was designed to use only youth‐reported measures, however due to data availability in the present study we selected parent‐reported measures for ‘non‐white ethnicity’, ‘traumatic events’, ‘loneliness’, ‘fights involvement’ and ‘ran away from home’. Finally, the use of a binary ethnicity variable–namely categories of “white” and “non‐white”–is a limitation of our study and IDEA‐RS as this reduction cannot capture the diversity of lived experiences among young people of marginalised ethnicities who may face disproportionate rates of mental health issues (Anderson & Mayes, 2010; del Río‐González, Holt, & Bowleg, 2021).

## CONCLUSION

Overall, the IDEA‐RS has proved broadly generalisable across countries and cultures, consistently performing better‐than‐chance. We highlight the need for refitting between samples for accuracy, which limits the clinical utility of this model as a risk stratification tool, as it would necessitate the existence of large, population‐representative cohort studies for adjusting the algorithm in each location. In conclusion, our results add to the body of evidence that the IDEA‐RS may be a valid tool for stratifying MDD risk in adolescents across cultures for research purposes.

## AUTHOR CONTRIBUTIONS

**Eileen Y. Xu**: Conceptualization; Data curation; Formal analysis; Investigation; Methodology; Resources; Validation; Visualization; Writing–original draft; Writing–review & editing. **Niamh MacSweeney**: Methodology; Supervision; Writing–review & editing. **Gladi Thng**: Writing–review & editing. **Miruna C. Barbu**: Conceptualization; Supervision; Writing–review & editing. **Xueyi Shen**: Writing–review & editing. **Alex S. F. Kwong**: Writing–review & editing. **Liana Romaniuk**: Writing–review & editing. **Andrew McIntosh**: Supervision; Writing–review & editing. **Stephen M. Lawrie**: Conceptualization; Supervision; Writing–review & editing. **Heather C. Whalley**: Conceptualization; Supervision; Writing–review & editing.

## CONFLICT OF INTEREST STATEMENT

The authors have no conflicts of interest to declare.

## ETHICAL CONSIDERATIONS

ABCD data collection sites obtained informed consent and assent from children and their parent(s)/legal guardian(s) consistent with guidance from each site’s institutional review board. The authors accessed de‐identified ABCD data through a signed NIMH Data Archive Data Use Certification (DUC).

## IRB STATEMENT

ABCD data collection sites obtained informed consent and assent consistent with guidance from each site’s institutional review board. The authors accessed de‐identified ABCD data through a signed NIMH Data Archive Data Use Certification (DUC).

## Supporting information

Supporting Information S1

## Data Availability

Data used in the preparation of this article were obtained from the Adolescent Brain Cognitive Development^®^ (ABCD) Study (https://abcdstudy.org), held in the NIMH Data Archive (NDA). This is a multisite, longitudinal study designed to recruit more than 10,000 children age 9–10 and follow them over 10 years into early adulthood.
