# Supplementary material for: Assessing a prediction model for depression risk using an early adolescent sample with self‐reported depression
Source: JCPP Adv. 2024 Sep 3;5(2):e12276. doi: 10.1002/jcv2.12276 (PMC12159326; doi:10.1002/jcv2.12276)
Supplement: Supplementary file 1 — Supporting Information S1 [file JCV2-5-e12276-s001.docx]

**Assessing a prediction model for depression risk using an early adolescent sample with self-reported depression**

**Supplementary Materials**

**Coding MDD from KSADS**

MDD cases were coded on the following criteria:

“Impairment in functioning due to depression”

PLUS at least 1 core symptom and at least 5 total symptoms.

Core symptoms:

- Depressed mood AND/OR hopeless AND/OR irritability
- Anhedonia

Secondary symptoms:

- Any of: Weight Gain, Weight Loss, Increased Appetite, Decreased Appetite
- Any of: Insomnia when depressed, Hypersomnia
- Any of: Psychomotor Agitation in Depressive Disorder, Psychomotor Retardation
- Fatigue
- Any of: Guilt, Decreased Self-Esteem
- Any of: Concentration Disturbance, Indecision
- Any suicidality (ksads_23)

**Selecting a penalty factor for model refitting using Penalised Maximum Likelihood Estimation**

Analyses were conducted in R version 4.4.0. The val.prob() function from the R package ‘rms’ was used to extract the penalty which optimized model AICc. Penalty selection was conducted over 1000 bootstraps with resampling in a two-step process:

First, we tested a set of 50 penalty terms ranging from 10^-2 to 10^5 to estimate the magnitude of optimal penalties. For self-reported incident depression, number% of converged models had penalties between 0 and 10. To fine-tune the penalty term, we then tested a set of 100 penalty terms ranging from 0.01 to 10. We observed ceiling effects in this second set of penalty terms which did not improve upon increasing the range of penalties - thus, we elected to use the median penalty value across 1,000 bootstraps for self-reported model refitting.

The same process was taken for parent-reported model refitting. We began with a set of 50 penalty terms ranging from 10^-2 to 10^5 for parent-reported incident MDD. 99% of bootstrapped iterations gave penalties ranging from 0.01 to 10. Next, we tested a set of 100 penalties evenly split across this range. Ceiling effects were less severe for parent-reported incident MDD. Consistent with the self-reported model, we selected the median penalty value across 1,000 bootstrapped penalties.

**Overlap between youth-reported and parent-reported depression phenotypes**

Table S1: Overlap between youth-reported and parent-reported incident MDD in the large sample of unrelated individuals only (N = 9,854)

|  | Youth-reported incident MDD | | |
| --- | --- | --- | --- |
| Parent-reported incident MDD | Case (N = 68) | Control (N = 8,430) | Missing (N = 1,356) |
| Case (N = 52) | 3 (4.41%) | 44 (0.52%) | 5 (0.37%) |
| Control  (N = 8,511) | 60 (88.24%) | 8,357 (99.13%) | 94 (6.93%) |
| Missing  (N = 1,291) | 5 (7.35%) | 29 (0.34%) | 1,257 (92.70%) |

Table S2: Overlap between youth-reported and parent-reported increased DS in the large sample of unrelated individuals only (N = 9,854)

|  | Youth-reported increased DS | | |
| --- | --- | --- | --- |
| Parent-reported increased DS | Case (N = 365) | Control (N = 8,239) | Missing (N = 1,250) |
| Case (N = 373) | 42 (11.51%) | 331 (4.02%) | 0 (0%) |
| Control  (N = 8,231) | 323 (88.49%) | 7,908 (95.98%) | 0 (0%) |
| Missing  (N = 1,250) | 0 (0%) | 0 (0%) | 1,250 (100%) |

**Parent-report sensitivity analyses**

Table S3: Descriptive statistics for ABCD data by parent-reported incident MDD (Total N = 7,767)

| Demographics | | | | Control (N=7,722) | Case (N = 45) |
| --- | --- | --- | --- | --- | --- |
| Age at baseline assessment | | | | 9.9 (0.61) | 10.07 (0.65) |
| Age at 2-year follow-up | | | | 11.99 (0.65) | 12.23 (0.67) |
| Household income: | | <50K | | 1890 (26.5%) | 14 (33.3%) |
| ≥50K & <100K | | | | 2072 (29.1%) | 20 (47.6%) |
| ≥100K | | | | 3165 (44.4%) | 8 (19.0%) |
| Missing data | | | | 595 (7.7%) | 3 (6.7%) |
| Parental education: | | <HS Diploma | | 320 ( 4.1%) | 3 ( 6.7%) |
| HS Diploma/GED | | | | 651 ( 8.4%) | 3 ( 6.7%) |
| Some College | | | | 1903 (24.7%) | 13 (28.9%) |
| Bachelor | | | | 2038 (26.4%) | 10 (22.2%) |
| Postgraduate Degree | | | | 2801 (36.3%) | 16 (35.6%) |
| Missing data | | | | 9 (0.1%) | 0 (0.0%) |
| IDEA-RS Predictors | | | | | |
| Female sex | | | | 3661 (47.4%) | 20 (44.4%) |
| Ethnicity: | Black/African American | | | 966 (12.5%) | 5 (11.1%) |
| Hispanic/Latino | | | | 1166 (15.1%) | 4 ( 8.9%) |
| White/Caucasian | | | | 4340 (56.2%) | 31 (68.9%) |
| Other* | | | | 1250 (16.2%) | 5 (11.1%) |
| Traumatic events: | | | One | 1986 (25.7%) | 9 (20.0%) |
|  | | | Multiple | 677 ( 8.8%) | 11 (24.4%) |
| School disengaged^1^ | | | | 485 ( 6.3%) | 7 (15.6%) |
| Complains of loneliness^2^ | | | | 924 (12.0%) | 22 (48.9%) |
| Gets in fights^3^ | | | | 260 ( 3.4%) | 9 (20.0%) |
| Runs away from home^4^ | | | | 27 ( 0.3%) | 4 ( 8.9%) |
| Ever tried alcohol/cigarettes/drugs^5^ | | | | 1800 (23.3%) | 12 (26.7%) |
| Relationship with caregiver 1 | | | Q1 – Best | 1607 (20.8%) | 5 (11.1%) |
| Q2 - Very good | | | | 1556 (20.2%) | 4 ( 8.9%) |
| Q3 – Good | | | | 1577 (20.4%) | 15 (33.3%) |
| Q4 – Poor | | | | 1511 (19.6%) | 7 (15.6%) |
| Q5 - Poorest | | | | 1471 (19.0%) | 14 (31.1%) |
| Relationship with caregiver 2 | | | Q1 - Best | 1550 (20.1%) | 9 (20.0%) |
| Q2 - Very good | | | | 1544 (20.0%) | 9 (20.0%) |
| Q3 – Good | | | | 1556 (20.2%) | 7 (15.6%) |
| Q4 – Poor | | | | 1539 (19.9%) | 7 (15.6%) |
| Q5 - Poorest | | | | 1533 (19.9%) | 13 (28.9%) |
| Family conflict | | | Q1 - Least | 1639 (21.2%) | 6 (13.3%) |
| Q2 - Little | | | | 1610 (20.8%) | 8 (17.8%) |
| Q3 - Moderate | | | | 1541 (20.0%) | 7 (15.6%) |
| Q4 - Much | | | | 1534 (19.9%) | 7 (15.6%) |
| Q5 - Most | | | | 1398 (18.1%) | 17 (37.8%) |

*Table S4: Fit statistics for replication and re-fitting of IDEA-RS in ABCD using parent-reported incident MDD and DS*

|  | Pelotas | | ABCD (external validation) | | ABCD (intercept recalibration) | | ABCD (fully refitted) | |
| --- | --- | --- | --- | --- | --- | --- | --- | --- |
|  | Incident MDD | Increase DS | Incident MDD | Increase DS | Incident MDD | Increase DS | Incident MDD | Increase DS |
| AUC (95% CI) | 77.8% (75.6% - 79.9%) | - | 63.12% (54.8% - 71.6%) | 56.5% (53.2% - 59.8%) | 63.2% 54.8% - 71.6%] | 56.5% (53.2% - 59.8%) | 86.9% (81.9% - 91.9%) | 61.1% (57.8% - 64.4%) |
| Brier score | 0.03 | - | 0.006 | 0.04 | 0.006 | 0.04 | 0.005 | 0.04 |
| Calibration in-the-large | 0.00 | - | -2.70 | -1.70 | -1.79 | -1.17 | 0.47 | -1.15 |
| Calibration slope | 1.26 | - | 0.64 | 0.37 | 0.64 | 0.37 | 1.11 | 0.35 |

MDD = Major Depressive Disorder. DS = Depression Symptoms. ABCD = Adolescent Brain Cognitive Development study. IDEA-RS = Identifying Depression in Early Adolescence Risk Score.

AUC: area under the curve, equivalent to C-statistic for binary outcomes. Values closer to 100% indicate better discriminative accuracy.

Brier score: quadratic scoring rule that combines calibration and discrimination; a score of 0 indicates perfect overall fit.

Calibration in-the-large: model intercept; results closer to 0 indicate better overall agreement between predicted probabilities and observed probabilities.

Calibration slope: values <1 indicate overly extreme model predictions, values >1 indicate the opposite. A calibration slope of 1 indicates perfect calibration.

Figure S1: Calibration curves for IDEA-RS prediction of incident MDD and increased DS (Self-reported)


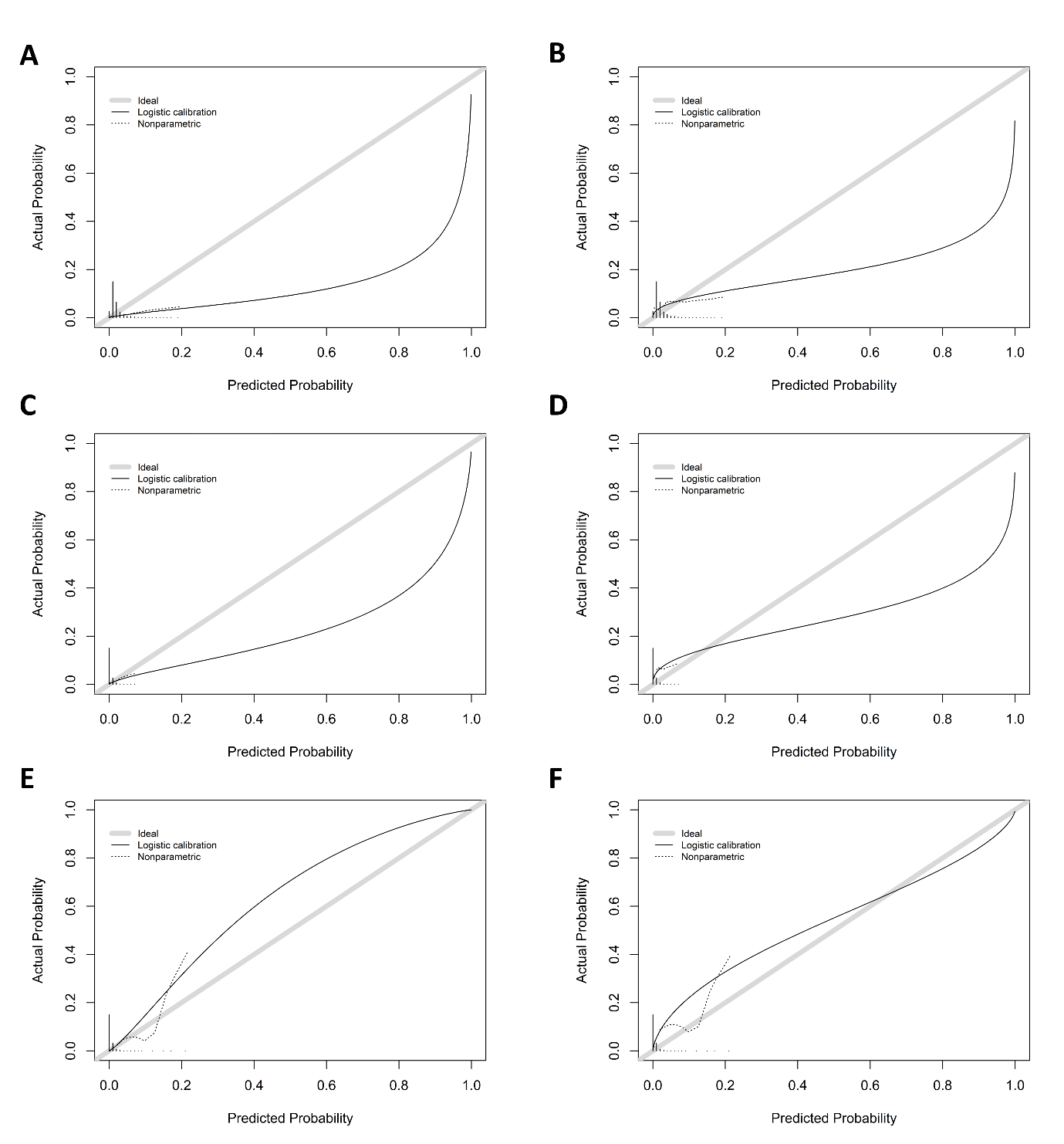


Figure S1: Calibration curves for (A) external replication of IDEA-RS in ABCD predicting incident MDD; (B) external replication predicting increased DS; (C) ABCD recalibrated model predicting incident MDD; (D) ABCD recalibrated model predicting increased DS; (E) ABCD refitted model predicting incident MDD, (F) ABCD refitted model predicting increased DS. The 45-degree diagonal line indicates perfect calibration; a curve closer to the bottom-right corner indicates a systematic overestimation of risk (lows are too low, highs are too high) and vice versa.

Figure S2: ROC curves for external replication of IDEA-RS in ABCD (Parent-reported)


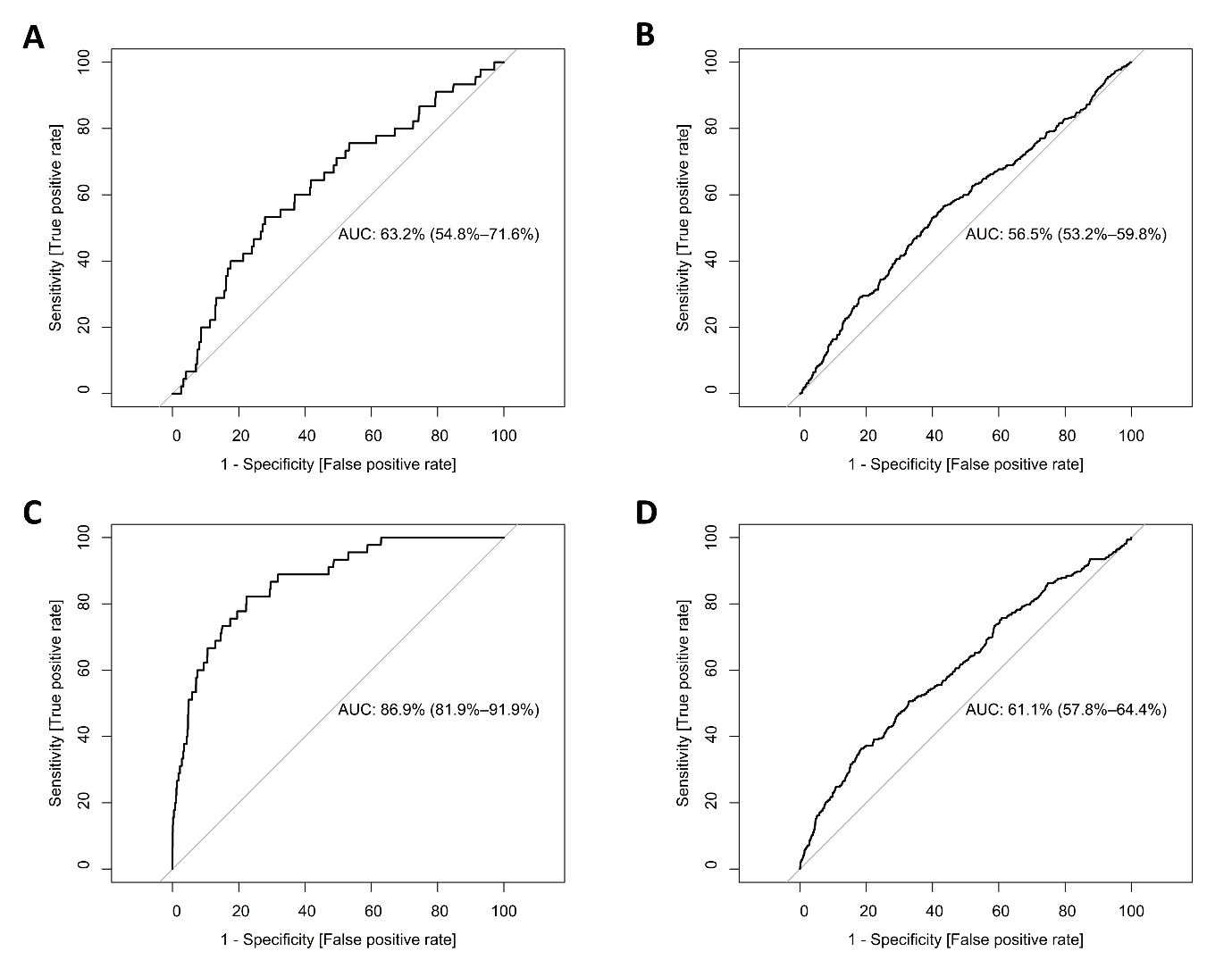


Figure S2: Receiver operating characteristic (ROC) curves for (A) external replication of IDEA-RS in ABCD with incident MDD as an outcome; (B) external replication with increased DS as an outcome; (C) ABCD re-fitted model of incident MDD as an outcome and (D) ABCD re-fitted model with increased DS as an outcome. The 45-degree diagonal indicates a classifier with no predictive value; classifiers with near-perfect discrimination are indicated by curves close to the top-left corner. AUC=Area Under the Curve. ABCD = Adolescent Brain Cognitive Development study. IDEA-RS = Identifying Depression in Early Adolescence Risk Score.

Figure S3: Calibration curves for IDEA-RS prediction of incident MDD and increased DS (Parent-reported)


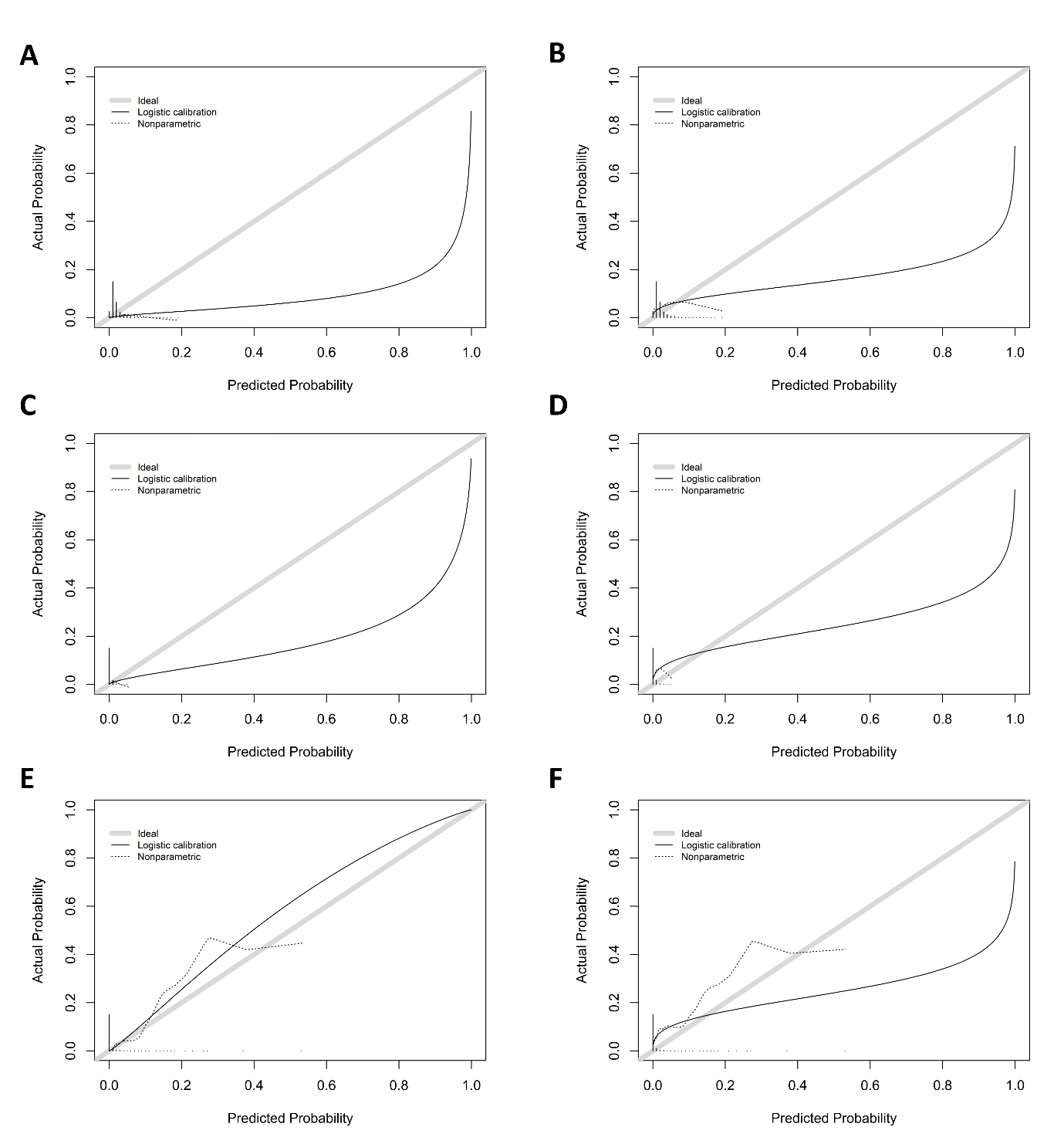


Figure S3: Calibration curves for (A) external replication of IDEA-RS in ABCD predicting incident MDD; (B) external replication predicting increased DS; (C) ABCD recalibrated model predicting incident MDD; (D) ABCD recalibrated model predicting increased DS; (E) ABCD refitted model predicting incident MDD, (F) ABCD refitted model predicting increased DS. The 45-degree diagonal line indicates perfect calibration; a curve closer to the bottom-right corner indicates a systematic overestimation of risk (lows are too low, highs are too high) and vice versa.

Figure S4: Predictor coefficients for IDEA-RS compared to re-fitted coefficients in ABCD (Parent-reported)


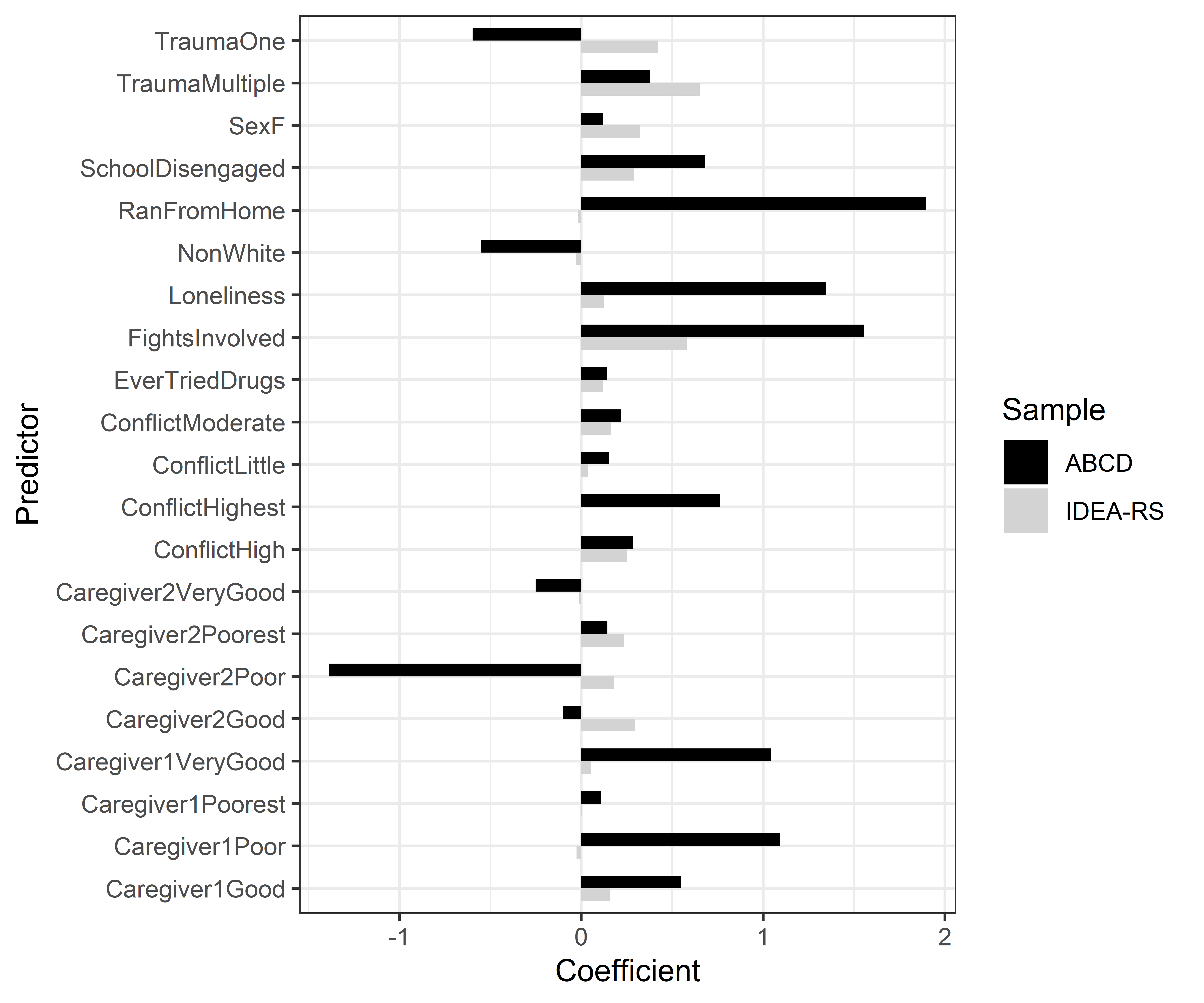


Figure S4: Coefficients from the re-fitted prediction model (ABCD re-fitted) compared to coefficients from IDEA-RS. Caregiver 1: primary caregiver. Caregiver 2: secondary caregiver. ABCD = Adolescent Brain Cognitive Development study. IDEA-RS = Identifying Depression in Early Adolescence Risk Score.
